# Vital Statistics

**Published:** 1886-02

**Authors:** 


**The Vital Statistics** for western New York show that the year 1885 has been unusually healthy. There has been a great reduction in deaths from scarlet fever, diarrhœal diseases, typhoid fever and zymotic diseases, as a class, with the exception of diptheria, which has been more fatal than in 1884. The death rate in Buffalo for 1885 was 19 per 1,000, a reduction of 1½ per 1,000 or 7½ per cent. from the preceding year. The causes for this reduction are, first, the cholera excitement which caused both the health authorities and the people generally to take extra precautions for the preservation of the public health.

